# Activities of Daily Living, Depression, and Quality of Life in Parkinson's Disease

**DOI:** 10.1371/journal.pone.0102294

**Published:** 2014-07-15

**Authors:** Blake J. Lawrence, Natalie Gasson, Robert Kane, Romola S. Bucks, Andrea M. Loftus

**Affiliations:** 1 Curtin Neuroscience Laboratory, School of Psychology and Speech Pathology, Curtin University, Perth, Western Australia, Australia; 2 Parkinson's Centre (ParkC), Curtin University, Perth, Western Australia, Australia; 3 School of Psychology, University of Western Australia, Perth, Western Australia, Australia; CSIR-Central Drug Research Institute, India

## Abstract

This study examined whether activities of daily living (ADL) mediate the relationship between depression and health-related quality of life (HR-QOL) in people with Parkinson's disease (PD). A cross-sectional, correlational research design examined data from 174 participants who completed the Geriatric Depression Scale (GDS-15), Parkinson's Disease Questionnaire-39 (PDQ-39), and Unified Parkinson's Disease Rating Scale-section 2 (UPDRS-section 2 [ADL]). Multiple Regression Analysis (MRA) was used to examine the mediator model. Depression and ADL significantly (*p*<.001) predicted HR-QOL, and depression significantly (*p*<.001) predicted ADL. Whilst ADL did not impact on the relationship between depression and HR-QOL, there was a significant (*p*<.001) indirect effect of depression on HR-QOL via ADL, suggesting both direct and indirect (via ADL) effects of depression on HR-QOL. The magnitude of this effect was moderate (*R* ^2^ = .13). People with PD who report depression also experience greater difficulty completing ADL, which impacts upon their HR-QOL. It is recommended that clinicians adopt a multidisciplinary approach to care by combining pharmacological treatments with psycho/occupational therapy, thereby alleviating the heterogeneous impact of motor and non-motor symptoms on HR-QOL in people with PD.

## Introduction

In addition to the cardinal motor symptoms of Parkinson's disease (PD), approximately 42% of people with PD report depressive symptoms [1]. Research suggests that comorbid depression in PD adversely impacts both health-related quality of life (HR-QOL) and the capacity to complete activities of daily living (ADL) [2].

Poor motor function, body discomfort and pain are common consequences of PD, which can lead to social isolation and negatively impact HR-QOL [3]. Depression is also a strong predictor of poor HR-QOL in PD [4]. Schrag et al. [5] examined the impact of depression on HR-QOL in PD and found those with high levels of depression demonstrated significantly lower HR-QOL, compared to participants without depression. Likewise, Quelhas and Costa [4] and Carod-Artal et al. [6] also found that depressed participants had poorer HR-QOL compared to non-depressed participants. Indeed, depression may be a more significant predictor of HR-QOL in PD than motor symptoms [5].

In an examination of the relationship between motor (e.g., tremor) and non-motor (e.g., depression) symptoms and HR-QOL in PD, Qin et al. [7] found that non-motor symptoms accounted for 62% of the variance in HR-QOL scores, compared to motor symptoms which accounted for just 19%. A recent systematic review by Soh et al. [8] of the most significant contributors to HR-QOL in PD confirmed the importance of depression. Nineteen of the 29 studies included examined the impact of depression on HR-QOL, and all found that depression was the greatest significant predictor of HR-QOL.

Activities of Daily Living (ADL) are difficult for people with PD [9]. There is, however, limited research into the relationship between ADL and depression in PD [2]. Papapetropoulos et al. [10] examined whether there were any significant differences between the ADL of depressed and non-depressed people with PD. Participants who were depressed scored significantly worse on measures of ADL than those not depressed [10]. Piccinni et al. [11] examined the relationship between depression in people with PD and their degree of functional disability. They found that participants with high levels of depression (severe or moderate) scored significantly worse on ADL measures than participants with low levels of depression (mild). Finally, Dissanayaka et al. [2] reported a stronger negative relationship between impaired ADL and depression than the severity of motor symptoms in PD. These results suggest that as the severity of depressive symptoms increases, people with PD experience greater difficulty completing ADL regardless of the severity of their motor symptoms.

In addition to the relationship between ADL and depression, research has identified a significant relationship between ADL and HR-QOL [8], [12]. In Soh et al.'s [8] systematic review, impairment in daily functioning was identified in eight studies as a significant predictor of HR-QOL in PD. Rahman et al. [13] examined the impact of PD symptoms on the HR-QOL of people with PD. ADL significantly predicted HR-QOL and depression accounted for a significant 40.8% of the variance in HR-QOL [13]. These results indicate that ADL and depression independently predict poorer HR-QOL [13]. Kleiner-Fisman et al. [14] examined the correlation between specific PD symptoms and predictors of HR-QOL. As with depression, the strongest relationship was between ADL and HR-QOL, suggesting that the loss of independence and inability to complete ADL may also be more important determinants of HR-QOL than the motor symptoms of PD [14]. Taken together, these studies provide convincing evidence that depressive symptoms and the inability to complete ADL independently significantly impact upon HR-QOL in those with PD, beyond the impact of the severity of their motor symptoms.

Despite strong evidence that both depression and ADL are important determinants of HR-QOL in PD, and that depression leads to poorer ADL, no study to date has examined the impact of ADL and depression on HR-QOL. One possibility is that depression reduces ADL, which leads to poorer HR-QOL. That is, that the effect of depression on HR-QOL is mediated by ADL. Another is that both depression and ADL independently predict HR-QOL, and a final possibility is that depression has both direct and indirect effects on HR-QOL, in part via ADL.

The present study examined the relationship between depression and HR-QOL in people with PD. The following hypotheses were tested: (1) depression significantly predicts HR-QOL, (2) depression significantly predicts ADL, (3) ADL significantly predicts HR-QOL, and (4) ADL significantly mediates the relationship between depression and HR-QOL.

## Methods

### 1. Participants

People with idiopathic PD in Western Australia were invited to participate in the Parkinson's Centre (ParkC) research project located at Edith Cowan University. Participants were recruited through advertising, and referrals from neurologists and physicians. To be included in the study, participants must have been formally diagnosed with PD in accordance with the United Kingdom Parkinson's Disease Society Brain Bank Clinical Criteria (UKPDSBBC). Participants were excluded from the study if they scored below 24 on the Mini Mental Status examination (MMSE) [15]. The final sample included 174 participants (119 male), ranging in age from 41 to 85 years (*M* = 65.96, *SD* = 9.46). All participants provided written informed consent. Edith Cowan University's Research Ethics Committee approved this study.

### 2. Measures

The Geriatric Depression Scale-15 (GDS-15) is a self-report measure used to detect depression in older adults and contains 15 questions that require participants to answer “no” or “yes” and are summed to a total of 15 [16]. The GDS-15 is suitable for use in PD as it focuses on the social/psychological factors of depression and excludes the shared somatic symptoms of PD, thereby addressing problems of symptom overlap found with other measures [17]. The internal consistency (Cronbach's alpha) of the GDS-15 in the present study was α = .79.

The Parkinson's Disease Questionnaire-39 (PDQ-39) contains 39 items across eight dimensions of HR-QOL (mobility, activities of daily living [ADL], emotional well-being, stigma, social support, cognitions, communication, and bodily discomfort) [18]. Items are scored from “0 = never” to “4 = always (or cannot do at all)” and within each dimension the items are computed into a total score, by dividing the sum of question scores by the maximum score for that dimension and multiplying by 100 [18]. Each dimension ranges from “0 = no problem” to “100 = maximum level of problem” [18]. Higher scores indicate poorer HR-QOL. The PDQ-39 has been identified as a ‘recommended’ measure for use in PD for both clinical trials and epidemiological studies [19]. This study used a single index (SI) score by summing the dimension scores and dividing by eight. The SI score also ranges from “0 = no problem” to “100 = maximum level of problem” [18]. The internal consistency of the PDQ-39 in the present study was α = .93.

The UPDRS-section II (ADL) is the second subscale of the UPDRS, which assesses ADL among people with PD [20]. The UPDRS-section II (ADL) is completed by the researcher during an assessment and contains 13 items, each scored either “0 = normal”, “1 = slight”, “2 = mild”, “3 = moderate” or “4 = severe” difficulty with the task. Higher scores indicate greater severity of motor symptoms and greater interference with the participants' ability to complete ADL independently [21]. For the purposes of this study, a total score was calculated by summing the scores from the 13 items and dividing by 13. Internal consistency of the 13 items used in the present study was α = .84.

### 3. Procedure

Assessment times were scheduled during the participant's ‘on’ stage, approximately one hour post-medication. Each assessment was conducted by a trained researcher and took approximately 2.5 hours.

## Results

Independent samples *t*-tests revealed no significant gender differences for depression, ADL, and HR-QOL (*p*>.05). Bivariate correlations determined whether age significantly correlated with depression, ADL, and HR-QOL. Older age was associated with poorer ADL and was retained as a covariate (see Table 1).

**Table 1:** Descriptive statistics and correlations between study variables.

Variable	Mean	SD	1	2	3
1. Age	65.96	9.46	-		
2. Depression (max 15)	3.20	2.86	.05	-	
3. Activities of Daily Living (max 4)	.85	.53	.17*	.29**	-
4. Health-related Quality of Life (max 100)	19.25	12.44	.05	.63**	.60**

*Notes.*

*p<.05.

**p<.01.

*SD* = Standard deviation.

As predicted (Hypothesis 1), depression significantly correlated with HR-QOL and ADL. After controlling for the influence of age, depression remained a significant predictor of HR-QOL, accounting for an additional 23.3% of variance. Likewise, consistent with prediction (Hypothesis 3), ADL also significantly correlated with HR-QOL. Again, after controlling for age, ADL accounted for an additional and significant 19.2% of the variance in HR-QOL. The overall model of age, depression, and ADL was significant, *R* ^2^ = .59, adjusted *R* ^2^ = .59, *F* (3, 170) = 82.54, *p*<.001. Finally, and as predicted (Hypothesis 2) depression was a significant predictor of ADL after controlling for age, accounting for an additional 7.7% of the variance in ADL, *F* (1, 171) = 14.62, *p*<.001. Age and depression combined accounted for a significant 10.5% of the variance in ADL, *R* ^2^ = .11, adjusted *R* ^2^ = .10, *F* (2, 171) = 10.05, *p*<.001.

A Sobel test was used to test whether ADL had a significant indirect effect on the relationship between depression and HR-QOL [22]. The Sobel test result was significant, *z* = 3.58 (*p*<.001), indicating that ADL does have a significant, indirect effect on the relationship between depression and HR-QOL. Given the above relationships, hierarchical multiple regression analysis (MRA) was used to determine whether ADL mediates the relationship between depression and HR-QOL.

A final MRA tested whether this mediation effect was complete or partial. At Step 2, age and depression accounted for 40.1% of the variance in HR-QOL, *R* ^2^ = .40, adjusted *R* ^2^ = .39, *F* (2, 171) = 57.22, *p*<.001. ADL was entered at Step 3 and accounted for an additional 19.2% of the variance in HR-QOL, Δ*R* ^2^ = .19, *F* (1, 170) = 80.18, *p*<.001. Depression accounted for a significant 39.9% of the variance of HR-QOL at Step 2. However, when ADL was entered at Step 3, the amount of unique variance accounted for by depression reduced to 23.3% although this remained significant. Therefore, *partial mediation* can be inferred. Table 2 provides the MRA results.

**Table 2:** Hierarchical Regression Model Analyses for the Indirect Effect of Activities of Daily Living on Depression and Health-Related Quality of Life.

Predictor		*R* ^2^	β	B	Part Correlation
Step 1^a^		.03			
	Age		.17*	.01	.17
Step 2^a^		.11**			
	Age		.16*	.01	.16
	Depression		.28**	.05	.28
Step 1^b^		.00			
	Age		.05	.06	.05
Step 2^b^		.40**			
	Age		.02	.02	.02
	Depression		.63**	2.75	.63
Step 3^b^		.59**			
	Age		.−.06	−.075	−.06
	Depression		.50**	2.19	.48
	Activities of Daily Living		.46**	10.82	.44

*Notes.*

* *p*<.05;

**p<.001.

*R* ^2^ = *R* Square; β = Standardised beta coefficient; *B* = Unstandardised B coefficient; Part Correlation = Unique variance of each predictor;

a = Dependent variable is activities of daily living;

b = Dependent variable is health-related quality of life.

To declare partial mediation the change in the effect of depression on HR-QOL from Steps 2 to 3 must be a *significant* change [23]. A *z* test was conducted using the unstandardized beta coefficients and corresponding standard error values to determine the significance of the indirect effect of ADL [23]. A value greater than 1.96 at α = .05 is necessary for partial mediation [23]. The *z* test value of 1.65 indicates no significant difference between the two path coefficients. The change in depression when ADL was entered into the model was *not a significant* change, indicating that ADL does not partially mediate the relationship between depression and HR-QOL, thus hypothesis (4) was rejected. However, the Sobel test indicates that there remained a significant indirect effect of ADL on the relationship between depression and HR-QOL. Based on Cohen's [24] conventions the magnitude of this effect was moderate, *R* ^2^ = .13.

The previous analyses were repeated to examine whether shared variance between the UPDRS-section II (ADL) and the PDQ-39 (ADL-subscale) had any effect on the results. With the PDQ-39 (ADL-subscale) removed from the model, a significant Sobel test *z* = 3.40 (*p*<.001) indicated that ADL had a significant indirect effect on the depression and HR-QOL relationship [22]. MRA tested for complete or partial mediation. At Step 2, age and depression account for 42.6% (increase of 1.5%) of the variance in HR-QOL, *R* ^2^ = .43, adjusted *R* ^2^ = .42, *F* (2, 171) = 63.40, *p*<.001. ADL was entered at Step 3 and accounted for an additional 14.2% (decrease of 5%) of the variance in HR-QOL, Δ*R* ^2^ = .14, *F* (1, 170) = 55.65, *p*<.001. Depression accounted for a significant 42.5% (increase of 1.6%) of the variance in HR-QOL at Step 2. However, when ADL was entered at Step 3, the amount of unique variance accounted for by depression reduced to 27% (increase of 3.7%) although this remained significant. This result infers partial mediation.

With the PDQ-39 (ADL-subscale) is removed from the model, a *z* test returned a value of 1.40 to indicate that ADL does not partially mediate the relationship between depression and HR-QOL [23]. Nonetheless, the significant Sobel test indicates that ADL has a significant indirect effect on the relationship between depression and HR-QOL. The magnitude of this effect remained moderate, *R* ^2^ = .11 (decrease of .2) [24].

## Discussion

This is the first study to examine whether ADL mediates the relationship between depression and HR-QOL in people with PD. ADL demonstrated a signficant indirect effect on the depression and HR-QOL relationship, but did not reduce the relationship such that a partial mediation effect could be declared [23]. Although this result did not support hypothesis (4), the size of the indirect effect was moderate and suggests that people with PD with depressive symptoms also experience greater difficulty completing ADL, which consequently impacts upon their HR-QOL.

There are several implications from the present finding. Firstly, it may be valuable for clinicians to consider the non-motor factors relating to HR-QOL in people with PD [13]. Assessment of depressive symptoms and ADL in people with PD would provide a clearer understanding of these non-motor relationships and promote a multidisciplinary approach to care [8], [13]. A multidisciplinary approach combining pharmacological management with psycho/occupational therapy could alleviate the heterogeneous impact of motor and non-motor symptoms on HR-QOL in people with PD [13], [25], [26].

Secondly, the present findings highlight the complex interaction between non-motor symptoms and daily functioning in PD. Previous research has demonstrated that people with PD with more knowledge of the disease course are less likely to be depressed [2]. Educating people with PD about the impact of depression and impaired ADL may help them to better manage their symptoms, potentially reducing the likelihood of depression, and improving their HR-QOL [27].

Finally, the present findings suggest that depression and impaired ADL in combination lead to worse HR-QOL than the impact of the two independently. Therefore, psychotherapy interventions (e.g., CBT) for people with PD and depression may have additional benefits for those who also experience difficulty with ADL. Cognitive-behavioural therapy (CBT) can promote acceptance of assistance by a caregiver to provide help with ADL and combat feelings of dependence and helplessness for people with PD [27]. The CBT framework can include caregivers and family members as part of the treatment for the person with PD [27]. CBT teaches caregivers and family members techniques for supporting the person with PD, enabling them to respond positively when feelings of helplessness, dependence and depression are experienced by the person with PD [27].

In addition to the significant indirect effect, depression was the strongest predictor of HR-QOL (Hypothesis 1). This result is consistent with findings by Schrag et al. [3] and Qin et al. [7]. Previous studies have also reported that people with PD and depression score significantly worse on measures of HR-QOL than people with PD without depression [6]. The present findings confirm that depression is a significant determinant of HR-QOL for Western Australian people with PD.

Supporting previous research by Dissanayaka et al. [2], depression significantly predicted ADL, whereby participants with depression reported greater impairment in ADL (Hypothesis 2). The significant relationship between depression and ADL was previously reported Piccinni et al. [11], who found that impairment in ADL increased as the severity of depression increased.

ADL accounted for a significant proportion of variance in HR-QOL (Hypothesis 3). Kleiner-Fisman et al. [14] reported a similar significant relationship between ADL and HR-QOL for people with PD, whereby impaired ADL was associated with poorer HR-QOL. As motor symptoms progress, people with PD experience difficulty completing simple tasks such as using eating utensils, dressing, and walking [10]. These diffiulties adversely impact their HR-QOL [8]. The present study used a total index score for ADL; which can limit the ability to distinguish between the impact of specific ADL on HR-QOL. However, the present findings suggest that participants who reported difficulty completing ADL also reported a poorer HR-QOL.

The present results should be considered within some limitations. The participants' mean score for depressive symptoms was below the diagnostic cut off for depression, and the mean scores for ADL and HR-QOL were within the ‘normal’ to ‘slightly impaired’ range [17], [18], [21]. This limited degree of impairment and symptom severity reduces the generalisability of the findings to the PD population [28]. Depression and impaired HR-QOL have been found in early stages of PD [28]. In addition, the prevalence of comorbid minor and major depression has been reported at 22% and 17% [29]. This relatively unimpaired sample reflects the early stage of PD onset of these participants, which may have contributed to the lack of partial mediation. Lastly, due to the cross-sectional design of this study, it is difficult to determine the temporal relationship between depression, ADL, and HR-QOL.

The novel nature of the indirect effect of ADL on the depression and HR-QOL relationship in PD provides opportunities for future research. Recruitment of a sample that captures the spectrum of symptom severity would provide a more accurate representation of the heterogeneity in PD and increase both the external validity of the results and the possibility of finding mediation effects [30]. Furthermore, a longitudinal investigation gathering depression scores at Time 1, ADL scores at Time 2, and HR-QOL scores at Time 3, would allow causal inferences to be made when interpreting a mediation effect.

In conclusion, this is the first study to demonstrate a significant indirect effect of ADL on the depression and HR-QOL relationship in PD. The moderate effect size suggests that people with PD and depressive symptoms experience greater difficulty completing ADL, which adversely impacts upon their HR-QOL. It is recommended that clinicians adopt a multidisciplinary approach when caring for people with PD and consider the relationships between depression, ADL, and HR-QOL. Undoubtedly, this significant effect warrants further investigation and accentuates the relationship between depression and HR-QOL in PD.

## References

[1] Ishihara L, Brayne C (2006) A systematic review of depression and mental illness preceding Parkinson's disease. Acta Neurol Scand 113: 211–220. 16542159 10.1111/j.1600-0404.2006.00579.x

[2] Dissanayaka NNW, Sellbach A, Silburn PA, O'Sullivan JD, Marsh R, et al (2011) Factors associated with depression in Parkinson's disease. J Affect Disord 132: 82–88. 21356559 10.1016/j.jad.2011.01.021

[3] Schrag A (2006) Quality of life and depression in Parkinson's disease. J Neurol Sci 248: 151–157. 16797028 10.1016/j.jns.2006.05.030

[4] Quelhas R, Costa M (2009) Anxiety, depression, and quality of life in Parkinson's disease. The J Neuropsychiatry Clin Neurosci 21: 413–419. 19996250 10.1176/jnp.2009.21.4.413

[5] Schrag A, Jahanshahi M, Quinn N (2000) What contributes to quality of life in patients with Parkinson's disease? J Neurol Neurosurg Psychiatry 69: 308–312. 10945804 10.1136/jnnp.69.3.308 PMC1737100

[6] Carod-Artal FJ, Ziomkowski S, Mourão MH, Martínez-Martin P (2008) Anxiety and depression: main determinants of health-related quality of life in Brazilian patients with Parkinson's disease. Parkinsonism Relat Disord 14: 102–108. 17719828 10.1016/j.parkreldis.2007.06.011

[7] Qin Z, Zhang L, Sun F, Fang X, Meng C, et al (2009) Health related quality of life in early Parkinson's disease: impact of motor and non-motor symptoms, results from Chinese levodopa exposed cohort. Parkinsonism Relat Disord 15: 767–771. 19553154 10.1016/j.parkreldis.2009.05.011

[8] Soh SE, Morris ME, McGinley JL (2011) Determinants of health-related quality of life in Parkinson's disease: a systematic review. Parkinsonism & Relat Disorders 17: 1–9. 10.1016/j.parkreldis.2010.08.012 20833572

[9] Wichowicz HM, Sławek J, Derejko M, Cubała WJ (2006) Factors associated with depression in Parkinson's disease: a cross-sectional study in a Polish population. Eur Psychiatry 21: 516–520. 16531018 10.1016/j.eurpsy.2006.01.012

[10] Papapetropoulos S, Ellul J, Argyriou AA, Chroni E, Lekka NP (2006) The effect of depression on motor function and disease severity of Parkinson's disease. Clin Neurol Neurosurg 108: 465–469. 16150537 10.1016/j.clineuro.2005.08.002

[11] Piccinni A, Marazziti D, Veltri A, Ceravolo R, Ramacciotti C, et al (2012) Depressive symptoms in Parkinson's disease. Compr Psychiatry 53: 727–731. 22209634 10.1016/j.comppsych.2011.11.002

[12] Carod-Artal FJ, Vargas AP, Martinez-Martin P (2007) Determinants of quality of life in Brazilian patients with Parkinson's disease. Mov Disord 22: 1408–1415. 17516479 10.1002/mds.21408

[13] Rahman S, Griffin HJ, Quinn NP, Jahanshahi M (2008) Quality of life in Parkinson's disease: the relative importance of the symptoms. Mov Disord 23: 1428–1434. 18543333 10.1002/mds.21667

[14] Kleiner-Fisman G, Stern MB, Fisman DN (2010) Health-related quality of life in Parkinson disease: correlation between health utilities index III and unified Parkinson's disease rating scale in U.S. male veterans. Health Qual Life Outcomes 8: 1–9. 20799993 10.1186/1477-7525-8-91 PMC2939643

[15] Folstein MF, Folstein SE, McHugh PR (1975) “Mini-mental state”: a practical method for grading the cognitive state of patients for the clinician. J Psychiatr Res 12: 189–198. 1202204 10.1016/0022-3956(75)90026-6

[16] Yesavage JA, Sheikh JI (1986) 9/Geriatric depression scale (GDS): recent evidence and development of a shorter violence. Clin Gerontol 5: 165–173.

[17] Schrag A, Barone P, Brown RG, Leentjens AF, McDonald WM, et al (2007) Depression rating scales in Parkinson's disease: critique and recommendations. Mov Disord 22: 1077–1092. 17394234 10.1002/mds.21333 PMC2040268

[18] Peto V, Jenkinson C, Fitzpatrick R (1998) PDQ-39: a review of the development, validation and application of a Parkinson's disease quality of life questionnaire and its associated measures. J Neurol 245: 10–14. 10.1007/pl00007730 9617716

[19] Martinez-Martin P, Jeukens-Visser M, Lyons KE, Rodriguez-Blazquez C, Selai C, et al (2011) Health-related quality-of-life scales in Parkinson's disease: critique and recommendations. Mov Disord 26: 2371–2380. 21735480 10.1002/mds.23834

[20] Goetz CG, Tilley BC, Shaftman SR, Stebbins GT, Fahn S, et al (2008) Movement disorder society-sponsored revision of the unified Parkinson's disease rating Scale (MDS-UPDRS): scale presentation and clinimetric testing results. Mov Disord 23: 2129–2170. 19025984 10.1002/mds.22340

[21] Martínez-Martín P, Benito-León J, Alonso F, Catalán MJ, Pondal M, et al (2003) Patients', doctors', and caregivers' assessment of disability using the UPDRS-ADL section: are these ratings interchangeable? Mov Disord 18: 985–992. 14502665 10.1002/mds.10479

[22] Baron RM, Kenny DA (1986) The moderator-mediator variable distinction in social psychology research: conceptual, strategic, and statistical consideration. J Pers Soc Psychol 6: 1173–1182. 10.1037//0022-3514.51.6.1173 3806354

[23] Frazier PA, Tix AP, Barron KE (2004) Testing moderator and mediator effects in counseling psychology research. J Counsel Psychol 51: 115–134..

[24] Cohen J (1988) Statistical Power Analysis for the Behavioural Sciences. 2nd Ed. New Jersey: Lawrence Erlbaum Associates.

[25] Keus SHJ, Bloem BR, Hendriks EJM, Bredero-Cohen AB, Munneke M (2007) Evidence-based analysis of physical therapy in Parkinson's disease with recommendations for practice and research. Mov Disord 22: 451–460. 17133526 10.1002/mds.21244

[26] Meek C, Morgan E, Walker MF, Furmstom A, Aragon A, et al (2010) Occupational therapy to optimise independence in Parkinson's disease: the designing and recording of a randomised controlled trial intervention. Br J Occup Ther 73: 178–185.

[27] Dobkin RD, Menza M, Beinfair KL (2008) CBT for the treatment of depression in Parkinson's disease: a promising nonpharmacological approach. Expert Rev Neurother 8: 27–35. 18088199 10.1586/14737175.8.1.27 PMC2743417

[28] Lewis SJG, Foltynie T, Blackwell AD, Robbins TW, Owen AM, et al (2004) Heterogeneity of Parkinson's disease in the early clinical stages using a data driven approach. J Neurol Neurosurg Psychiatry 76: 343–348. 10.1136/jnnp.2003.033530 PMC1739569 15716523

[29] Reijnders JSAM, Ehrt U, Weber WEJ, Aarsland D, Leentjens AFG (2008) A systematic review of prevalence studies of depression in Parkinson's disease. Mov Disord 23: 182–189. 10.1002/mds.21803 17987654

[30] Pagonabarraga J (2010) Parkinson's disease: definition, diagnosis, and management. In: Verhagen K, Verhagen ML, editors. Encyclopedia of movement disorders. Oxford: Academic Press. pp. 405–412.

